# Bromodomain proteins as potential therapeutic targets for B-cell non-Hodgkin lymphoma

**DOI:** 10.1186/s13578-024-01326-1

**Published:** 2024-11-23

**Authors:** Dan Zou, Sitong Feng, Bowen Hu, Mengya Guo, Yan Lv, Rong Ma, Yuxin Du, Jifeng Feng

**Affiliations:** 1 The Affiliated Cancer Hospital of Nanjing Medical University, Jiangsu Cancer Hospital, Jiangsu Institute of Cancer Research, Nanjing, China; 2 Research Center for Clinical Oncology, Jiangsu Cancer Hospital, Jiangsu Institute of Cancer Research, The Affiliated Cancer Hospital of Nanjing Medical University, Nanjing, China

**Keywords:** Bromodomains, B-cell non-Hodgkin lymphoma, Bromodomain and extra-terminal domain, Epigenetics

## Abstract

**Background:**

B-cell non-Hodgkin lymphoma (B-NHL) is the most common type of lymphoma and is significantly heterogeneous among various subtypes. Despite of considerable advancements in treatment strategies for B-NHL, the prognosis of relapsed/refractory patients remains poor.

**Main text:**

It has been indicated that epigenetic dysregulation is critically associated with the pathogenesis of most hematological malignancies, resulting in the clinical targeting of epigenetic modifications. Bromodomain (BRD) proteins are essential epigenetic regulators which contain eight subfamilies, including BRD and extra-terminal domain (BET) family, histone acetyltransferases (HATs) and HAT-related proteins, transcriptional coactivators, transcriptional mediators, methyltransferases, helicases, ATP-dependent chromatin-remodeling complexes, and nuclear-scaffolding proteins. Most pre-clinical and clinical studies on B-NHL have focused predominantly on the BET family and the use of BET inhibitors as mono-treatment or co-treatment with other anti-tumor drugs. Furthermore, preclinical models of B-NHL have revealed that BET degraders are more active than BET inhibitors. Moreover, with the development of BET inhibitors and degraders, non-BET BRD protein inhibitors have also been designed and have shown antitumor activities in B-NHL preclinical models. This review summarized the mechanism of BRD proteins and the recent progress of BRD protein-related drugs in B-NHL. This study aimed to collect the most recent evidences and summarize possibility on whether BRD proteins can serve as therapeutic targets for B-NHL.

**Conclusion:**

In summary, BRD proteins are critical epigenetic regulatory factors and may be potential therapeutic targets for B-NHL.

## Introduction

Non-Hodgkin lymphoma (NHL), the most common hematological malignancy in the world [1, 2], mainly (about 86% of NHL) originates from B cells [3]. B-cell NHL (B-NHL) comprises over 40 main subtypes, such as diffuse large B-cell lymphoma (DLBCL), Burkitt lymphoma (BL), mantle cell lymphoma (MCL), marginal zone lymphoma (MZL) and follicular lymphoma (FL) [4, 5]. Despite of high heterogeneity among different subtypes, most B-NHL patients can be cured after standard treatment with CHOP (cyclophosphamide, doxorubicin, vincristine, and prednisone) chemotherapy with or without anti-CD20 antibody rituximab [5, 6]. However, 30 to 50% of cases are resistant to treatment or relapse after remission, and their prognoses are markedly poor [6, 7]. In recent years, great progress has been made in targeted therapy and immunotherapy [8–10]. These treatments are more selective than chemotherapy; however, they have rare adverse reactions associated with their targets [11, 12], which affects their safety and tolerability. Therefore, more effective and safer treatments for relapsed/refractory (R/R) B-NHL patients are urgently required.

**Fig. 1:**
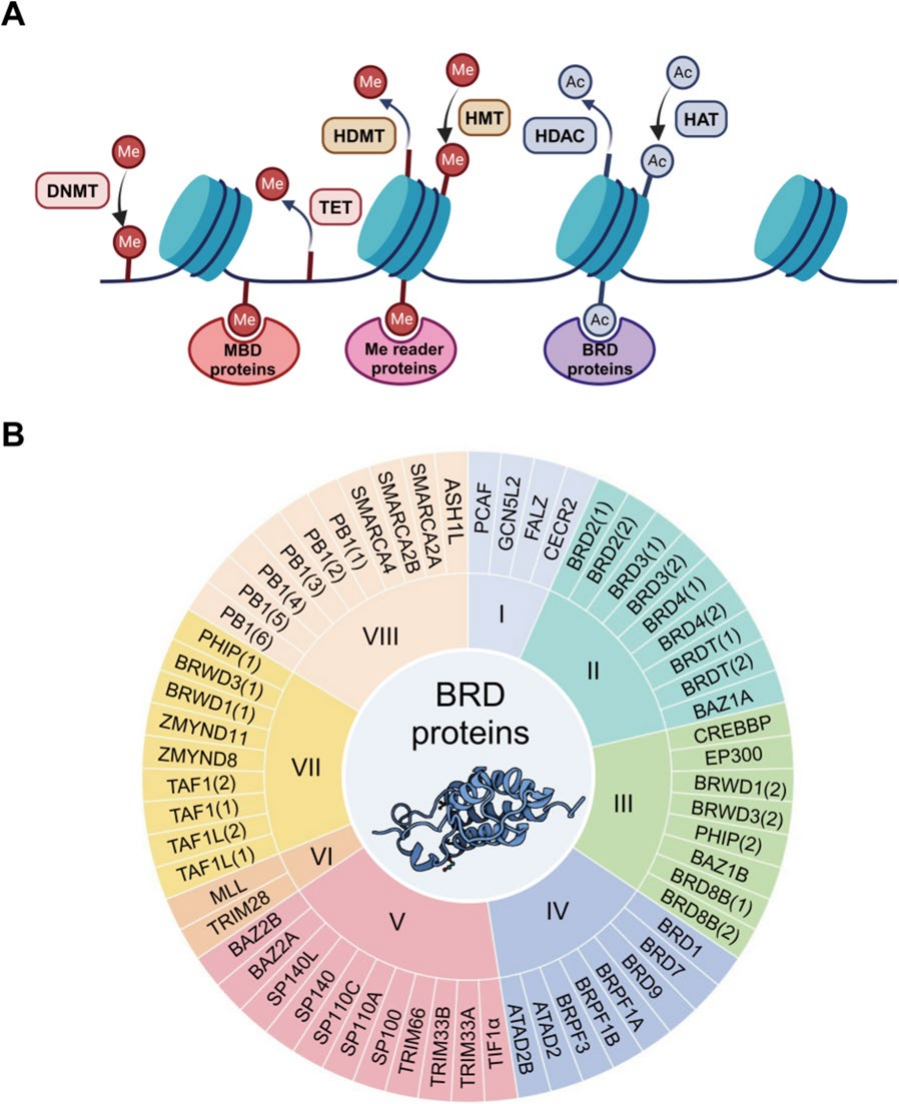
Mechanisms and types of common epigenetic enzymes. **A** Most common epigenetic modifications are histone acetylation/methylation and DNA methylation, which are mediated by enzymes namely ‘writers’, ‘erasers’ and ‘readers’ with examples of each given. **B** Different subfamilies of the human BRD proteins are named by Roman numbers (I–VIII). Center, the structure of BRD4(1) (PDB: 2OSS). Created with BioRender (http://www.biorender.com)

Epigenomics is a field that studies the covalent modifications of nucleic acids and histones, independent of the DNA sequence. Most common epigenetic modifications are histone acetylation/methylation and DNA methylation [13, 14]. The enzymes capable of depositing these chemical modifications are known as ‘writers’, while the enzymes that remove these modifications are defined as ‘erasers’. Furthermore, ‘readers’ are defined as the enzymes that recognize and dock at specific modifications already produced by ‘writers’ and ‘erasers’ [15, 16]. In terms of histone modifications, the writers contain enzymes such as histone methyltransferases (HMTs) and histone acetyltransferases (HATs), whereas the erasers mainly comprise histone demethylases (HDMTs) and histone deacetylases (HDACs), while the readers include bromodomains (BRDs), chromodomains (CDs), and so on. For DNA modifications, the writers, erasers, and readers primarily contain DNA methyltransferases (DNMTs), the ten-eleven translocation (TET) family, and methyl-CpG binding domains (MBDs), respectively (Fig. 1A) [16]. With the recent development of epigenomics, epigenetic dysfunction has been proved to be associated with the pathogenesis of most hematological malignancies; therefore, targeting epigenetics has become an essential strategy for the treatment of hematological malignancies [13, 14].

BRDs are a group of evolutionarily conserved protein domains that can recognize and bind acetylated lysine residues in histones, and function as the ‘readers’ [16]. BRDs comprise about 110 amino acids, and share a conserved fold with a left-hand bundle of four α helices (αC, αB, αA, and αZ) and two inter-helical loops (BC and ZA) [17]. In the human genome, 61 BRDs are distributed in 46 BRD proteins, which can be divided into eight subfamilies based on their structural or sequence similarity (Fig. 1B), including transcriptional coactivators (TRIM/TIF1), bromodomain and extra-terminal domain (BET) family (BRD4, BRD2, BRDT, BRD3), helicases (SMARCA), HATs and HAT-related proteins (GCN5, PCAF, CREBBP, EP300), transcriptional mediators (TAF1), methyltransferases (MLL, ASH1L), ATP-dependent chromatin-remodeling complexes (BAZ1B, BAZ1A), and nuclear-scaffolding proteins (PB1) [17–19]. Among these BRD proteins, the BET family is the most frequently researched subfamily in B-NHL. Based on the promising preclinical and clinical results of BET inhibitors (BETi)/degraders alone or combined with other anticancer drugs [20], non-BET BRD proteins and their related drugs have become a research focus [19]. This article reviewed the mechanism of BRD proteins in B-NHL and the development of BRD protein inhibitors/degraders for B-NHL treatment.

## Structure and mechanism of BRD proteins in B-NHL

### Bromodomain and extra-terminal domain family

**Fig. 2:**
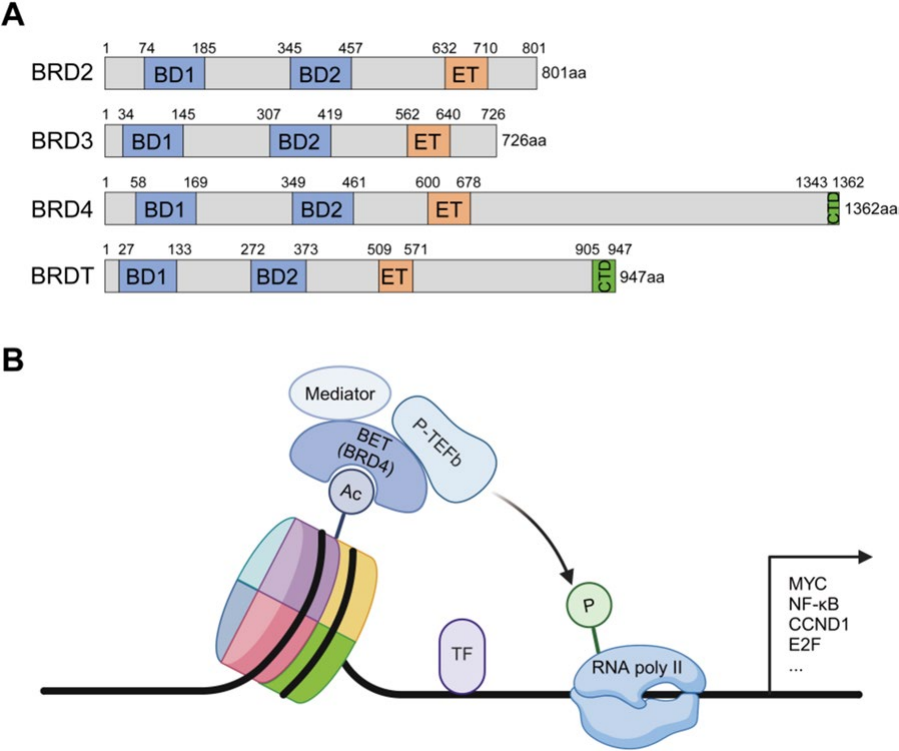
Domain structure and mechanism of the BET family. **A** Domain structure of human BET proteins BRD2, BRD3, BRD4, and BRDT, which contain two bromodomains (BD1 and BD2) and an extra-terminal domain (ET). BRD4 and BRDT also have a C-terminal domain (CTD). **B** Binding of BET proteins (such as BRD4) to acetylated histones recruits the positive transcription elongation factor b (P-TEFb) complex and mediates its activation. The activated P-TEFb phosphorylates and activates RNA polymerase II to initiate gene transcription. Created with BioRender (http://www.biorender.com)

The BET family members comprise BRDT, BRD2, BRD3, and BRD4, which contain two conserved N-terminal BRD modules (BD-1 and -2) and an extra-terminal domain (Fig. 2A). BRDT is primarily expressed in germ cells, while the other three are ubiquitously expressed [19]. BET proteins are important epigenetic regulators that induce the expression of various oncogenes in B-NHL (Fig. 2B). Mechanistically, through binding to the acetylated histones, BET proteins recruit the positive transcription elongation factor b (P-TEFb) complex and mediate its activation, leading to the phosphorylation and activation of RNA polymerase II to initiate gene transcription [19, 20].

The most frequent type of aggressive B-NHL is DLBCL, which primarily includes the activated B-cell (ABC) and germinal center B-cell (GCB) subtypes based on its cell origin [2]. Clinically, the ABC subtype is more aggressive, with reduced response to initial therapy and decreased overall survival [21]. The inhibition of BET proteins has been shown an activity against the transcription induced by super-enhancers, such as NF-κB-mediated transcription, which is essentially involved in ABC DLBCL. Mechanistically, BET proteins activated IKK via the MyD88/Toll-like receptor (TLR) and B-cell receptor (BCR) pathways, resulting in nuclear localization of NF-κB and transcription of its target genes [21]. In GCB DLBCL, chromosomal translocation of *MYC* is very common. Furthermore, BET proteins promoted the MYC expression, while BET inhibition reduced the MYC expression in DLBCL [22]. Moreover, BET proteins preferentially interacted with the enhancers crucial for determining B-cell fate, such as *PAX5* and *IRF8* [22]. Some BET-modulated genes were specific for certain histological subtypes; for instance, the *IRF4* gene was only observed in the ABC subtype but not in the GCB subtype [22]. In addition, BET proteins also modulated non-coding RNAs (such as miRNAs). In DLBCL cells, BETi was observed to downregulate several onco-miRNAs, such as miR-155-5p, miR-92a-1-5p, and miR-21-3p, as well as upregulate tumor-suppressing miRNAs, such as miR-16-5p and miR-96-5p [23].

BL, a highly aggressive form of B-NHL, is associated with distinctive rearrangements of the *MYC* oncogene, resulting in constitutive MYC overexpression [24]. The BET family also promoted MYC expression in BL, and BETi was found to reduce MYC expression and inhibit the proliferation of BL cell lines [25]. Therefore, based on the importance of MYC and BL, BET might be involved in the pathogenesis of BL.

MCL is a rare but aggressive form of B-NHL, accounting for 5–10% of all NHLs [26]. BRD4 has been reported to modulate the expression of several proteins involved in the cell cycle, including cyclin D1 and some cyclin-dependent kinases (CDKs), such as CDK4/6 [27]. This might be specifically associated with MCL, as its translocation at t(11;14)(q13;q32) fuses the *CCND1* and immunoglobulin heavy locus genes, causing overexpression of cyclin D1 and thus increasing CDK4/6 activity and accelerating the cell cycle progression [26]. Therefore, the BET family may be associated with MCL through the regulation of cyclin D1 and CDK4/6.

Epstein-Barr virus (EBV), discovered in 1964 [28], has been shown as a carcinogenic factor in the development of various lymphomas, such as BL and HL [29]. It has been indicated that BRD4 activated the EBV enhancer and promoter and modulated the expression of specific genes, such as EBV nuclear antigen 1 (EBNA1) [30]. EBNA1 is crucial for latent EBV infection through ensuring EBV genome replication and stable segregation, and activating the transcription of other genes associated with EBV latency. EBNA1 has been observed in all EBV^+^ tumors and is the only latent protein in certain tumors, such as latency I BL [29]. Altogether, the BET family may modulate different EBV life cycle stages and is associated with the development of EBV^+^ BL [31].

### CREBBP/EP300

CREBBP (CBP or KAT3A) and EP300 (p300 or KAT3B) are closely related HATs with many well-defined domains, including the KIX, CH1, CH3, and NCBD, which are associated with the catalytic core via long stretches of intrinsically disordered residues. The core comprises the CH2 region, BRD, and HAT domain [32].

FL, originating from germinal center (GC) B cells of the lymphoid follicle, is the most common indolent B-NHL but remains incurable, as 20% of patients relapse or progress after treatments [33]. Deregulation of epigenetic processes has been recognized as a central feature of FL, where epigenomic alterations have been observed in almost all patients [13]. The inactivating mutation of *CREBBP/EP300* is one of the most frequent genetic alterations in FL [34]. Most of the *CREBBP* mutations might promote lymphomagenesis through the control of antigen presentation and interferon signaling pathways, which were often associated with poor prognosis [35]. Moreover, deletion of *EP300* but not *CREBBP* partially reduced the number and size of GC, and their combined knockdown completely abrogated GC formation [34], indicating that the role of CREBBP and EP300 in modulating transcription in different functional and anatomic GC compartments is both common and distinct [34].

*CREBBP/EP300* mutations have also been observed in DLBCL and BL, which also originate from GCB cells [36]. Thus, this mutation might serve as a novel hallmark of GCB cell-originating lymphomas and indicate potential next-generation therapeutic targets for such malignancies [37].

In EBV associated lymphomas, EBNA leader protein (EBNALP) is among the first viral genes expressed after B-cell infection and is crucial for EBV-modulated immortalization of B-cell [38]. EBNALP has been observed to significantly enhance the transcription of EP300 after EP300 binds to the promoter region. Deletion of the EP300 HAT domain or the use of EP300 inhibitors abolished the co-activation of EBNALP and blocked the relationship between EP300 and EBNALP [39].

### BRD7/9

BRD7 was first identified as downregulated in nasopharyngeal carcinoma. Since then, decreased BRD7 activity has been associated with the pathophysiology of various diseases, such as obesity, diabetes, and cancers (e.g., breast, colorectal, hepatocellular, lung, and ovarian cancers) [40]. BRD7 has also been implicated in a variety of cellular mechanisms, mainly containing chromatin remodeling, transcriptional modulation, and cell cycle progression [40]. For instance, BRD7 negatively regulated the transcriptional activity and expression of baculoviral IAP repeat containing 2 (BIRC2), which functions as an oncogene in nasopharyngeal carcinoma [41]. Additionally, BRD7 was found to reduce the expression of cyclin D1 and downregulate the activity of Ras/Raf/MEK/ERK pathway, resulting in the inhibition of cell cycle progression and cell proliferation in hepatoma cells [42]. Whereas, in a study about EBV positive BL, host EBV latent infection upregulated BRD7, which then altered cellular and viral genome and enhanced EBV latency by interacting with EBNA1 to modulate MYC. However, the disruption of BRD7 reduced MYC expression and re-stimulated the lytic cycle, suggesting that BRD7 might inhibit EBV lytic reactivation. These data indicated that EBV can modulate the biphasic life cycle by interacting with host factors, including BRD7 [43].

BRD9 and BRD7 belong to BRD family IV, and their BRDs are highly homologous [17]. However, their association with tumor progression is quite different. Studies have indicated that BRD7 often acts as a tumor suppressor [40], whereas BRD9 is a pro-oncogenic protein [44], the mechanism of which is potentially linked with the STAT5 signaling pathway and the expression of MYC in hematological tumors [44, 45]. For example, BRD9 overexpression activated the STAT5 pathway, which is known to promote the proliferation and survival of AML cells [46]. BRD9 was also reported to support AML cell proliferation and an undifferentiated cell state by sustaining enhancer-mediated MYC expression using its bromodomain, and the inhibition of BRD9 bromodomain selectively suppressed the proliferation of human AML cell lines [47]. However, there are only a few studies on the relationship between BRD9 and B-NHL. Moreover, BRD9 mutation was only observed in Sézary syndrome, an aggressive leukemic form of cutaneous T cell lymphoma originating from the malignant transformation of central memory CD4^+^ T cells in the skin [48].

### SMARCA4

SMARCA4 (BRG1) and SMARCA2 (BRM) are essential subunits of SWI/SNF complexes which act as chromatin remodelers that change nucleosome structure [19]. *SMARCA4* loss-of-function mutations have been observed in different human cancers, such as lung, gastric, breast, ovarian, pancreatic, prostate, and hematological cancers [49]. Furthermore, SMARCA4 mutations are more frequent in BL, but less frequent in FL and DLBCL [37].

ARID1A is also a key subunit of SWI/SNF complexes. It has been reported that the complexes might block cell cycle progression and regulate the sensitivity to Fas-induced apoptosis in B-NHL [37]. Moreover, mutations in *ARID1A* and *SMARCA4* were found to render the essential areas of chromatin inaccessible to the master transcription factors that modulate GC reactions and cell fate decisions, thus causing B cells to aberrantly re-enter GC reactions and undergo repeated rounds of proliferation and somatic hypermutation. These findings provided a reasonable mechanism that mutations in *ARID1A* and *SMARCA4* would contribute to lymphomagenesis due to sustained cell division and ongoing mutation of B cells [50–52]. Overall, these data indicated that SMARCA4 may act as a tumor suppressor, and its loss-of-function mutation may promote cancer.

### Speckled protein (SP) family

The SP family members are components of promyelocytic leukemia nuclear bodies, including SP110, SP100, SP140L, and SP140 [19]. Furthermore, they contain a nuclear localization signal and various functional domains, for instance, a SAND domain (SP100, AIRE, NucP41/P75, and DEAF), which directly interacts with DNA or promotes protein–protein interactions, a BRD reading histone acetylation and a plant homeodomain (PHD) reading histone methylation [53].

SPs are highly expressed in mature and developing B-cells and act as interferon-stimulated genes [53]. For instance, SP100 expression is induced by interferons [54, 55], and interferons have been shown antiviral and anticancer activities [56, 57], thus, SP100 was reported to function as a tumor suppressor mechanistically through inhibiting the transcriptional activity of ETS1, which is important in several cellular processes such as proliferation, differentiation, invasion, angiogenesis and lymphoid development [54, 55]. In EBV positive BL cells, nuclear domain 10 was intact in the period of latent EBV infection, and became dispersed upon lytic activation, with SP100, Daxx, NDP55 dispersed before and PML dispersed after the onset of lytic replication [58]. Clinically, mutation of *SP140* was one of the most frequent variants in MCL patients at diagnosis or relapse [59] and was often linked with substandard prognosis [60]. In addition, compared to classical MCL, the frequency of mutation in *SP140* was much lower in indolent MCL [61].

### Transcriptional intermediary factor 1 (TIF1) family

TIF1 family proteins TIF1α (TRIM24), TIF1β (TRIM28), TIF1γ (TRIM33), and TIF1δ (TRIM66) are a subfamily of the tripartite-motif (TRIM) family. These proteins comprise a C-terminal PHD finger, a BRD, and an N-terminal TRIM domain [19].

TIF1 proteins have various functions, such as immunity, transcription, DNA repair, cell differentiation, and mitosis, all of which may be altered during tumorigenesis [62]. As reported, high TRIM28 expression was positively linked with substandard survival of B-NHL patients and served as an independent prognostic marker [63]. Furthermore, TRIM28 promoted B-NHL cell proliferation by regulating cell cycle progression, and suppression of TRIM28 expression increased the sensitivity to Bortezomib via the p53-mediated apoptosis pathway [63]. Altogether, TRIM28 may act as a tumor promoter in B-NHL and a novel target for Bortezomib resistance. Moreover, the literature has indicated that *SEPT6_TRIM33* gene fusion was significantly associated with poor PFS and was an independent prognostic factor for B-cell lymphoma [64].

The mechanism of non-BET BRD proteins is summarized in Table 1.

**Table 1:** Mechanism of non-BET BRD proteins in B-NHL

BRD protein	Possible mechanism	Diseases	Activity in tumors	References
CREBBP/EP300	Combined loss of CREBBP and EP300 completely abrogates GC formation	GCB-DLBCL, BL, FL	Tumor promoter	[34–36]
BRD7	Inhibiting tumorigenesis through chromatin remodeling, cell cycle progression, and transcriptional modulation	Breast, colorectal, lung, hepatocellular, and ovarian cancers	Tumor suppressor	[40]
BRD9	Activating the STAT5 signaling pathway and the MYC expression	AML	Tumor promoter	[44, 45]
SMARCA4	Loss of ARID1A and SMARCA4 contributes to lymphomagenesis by causing B cells to aberrantly re-enter germinal centers and undergo repeated rounds of proliferation and somatic hypermutation	DLBCL, BL, FL	Tumor suppressor	[50–52]
SP100	Acting as the interferon stimulated gene, and inhibiting the transcriptional activity of ETS1	Lymphomas	Tumor suppressor	[53–55]
TRIM28	Promoting cell proliferation, advancing cell cycle progression, and suppressing apoptosis pathway	B-NHL	Tumor promoter	[63]

## Preclinical activity of BRD protein inhibitors as single agents in B-NHL

Based on the mechanisms of BRD proteins in B-NHL, small molecule inhibitors targeting BRD proteins, especially the BET family, have been designed and researched. Preclinical B-NHL models have indicated that the effect of BETi mono-treatment or co-treatment with other anticancer drugs is significant. With the development of BETi, non-BET BRD protein inhibitors have also been studied. This section focuses on the preclinical studies on the use of BRD protein inhibitors monotherapy in B-NHL (Table 2).

**Table 2:** Preclinical studies on BRD protein inhibitors monotherapy in B-NHL

Compound	Type of agents	Diseases	In vitro/In vivo	Publication year	References
JQ1	BETi	BL	Both	2011	[25]
		DLBCL, BL, MCL	In vitro	2013	[65]
		ABC-DLBCL	In vitro	2014	[21]
		MCL	Both	2015	[66]
		KSHV + PEL	In vitro	2017	[70]
		DLBCL, BL	In vitro	2018	[67]
		DLBCL	In vitro	2018	[68]
		DHL/THL DLBCL, BL	In vitro	2019	[69]
		EBV + BL	In vitro	2021	[71]
OTX015	BETi	DLBCL	Both	2015	[72]
		MCL	Both	2018	[73]
		MCL	Both	2018	[74]
		DLBCL	Both	2018	[23]
		DHL/THL DLBCL, BL	In vitro	2019	[69]
		RT-DLBCL	In vitro	2021	[75]
CPI-203	BETi	ABC-DLBCL	Both	2014	[21]
		MCL	Both	2014	[76]
		DHL	Both	2018	[77]
		DLBCL, BL	In vitro	2018	[67]
		FL	Both	2024	[78]
CPI-0610	BETi	DLBCL	Both	2023	[79]
I-BET151	BETi	ABC-DLBCL	In vitro	2014	[21]
		MCL	Both	2015	[66]
		KSHV + PEL	In vitro	2017	[70]
		DLBCL, BL	In vitro	2022	[80]
I-BET762	BETi	DHL/THL DLBCL, BL	In vitro	2019	[69]
AZD5153	BETi	DLBCL, BL	In vitro	2022	[80]
ABBV-075	BETi	NHL	Both	2017	[81]
		RT-DLBCL	In vitro	2021	[75]
PFI-1	BETi	FL	Both	2024	[78]
INCB057643	BETi	DLBCL/HGBCL	In vitro	2021	[82]
PLX51107, PLX2853	BETi	DLBCL	Both	2020	[83]
YLT-LL-11	BETi	DLBCL	In vitro	2019	[84]
BAY1238097	BETi	DLBCL, MCL	In vitro	2017	[85]
RVX2135	BETi	Eμ-Myc Lymphoma	Both	2014	[86]
A-485	CBP/EP300 inhibitor	MCL	In vitro	2022	[87]
L002	EP300 inhibitor	Lymphoma	In vitro	2013	[88]
NEO2734	BET/CBP/EP300 inhibitor	DLBCL	Both	2020	[89]

### Preclinical studies on BETi monotherapy

Many in vitro and in vivo studies have been performed on JQ1, a competitive interactor of BRD2 and BRD4 acetyl-lysine binding motifs, to assess its antitumor effects in various B-NHL [21, 25, 65–69] (i.e., DLBCL, BL, MCL) and some virus-associated lymphomas, including Kaposi's sarcoma-associated herpes virus (KSHV) positive pleural effusion lymphoma (PEL) [70], and EBV positive BL [71]. As reported, its anti-proliferative effects on lymphomas were primarily through the induction of cell cycle arrest and apoptosis. Moreover, JQ1 might reduce MYC expression [25, 65, 67, 71] by downregulating proteins linked with MyD88/TLR, BCR, NF-κB, and JAK/STAT signaling pathways [21, 66].

The preclinical development of JQ1 has also led to the development of other BETi, structurally and functionally related to JQ1. Among them, CPI-203 in DLBCL, BL, MCL, FL models [21, 67, 76–78], CPI-0610 in DLBCL models [79], OTX015 (birabresib) in DLBCL, BL, MCL models [23, 69, 72, 74, 75], I-BET151 in DLBCL, BL, MCL, KSHV^+^ PEL models [21, 66, 70, 80], as well as I-BET762 [69] and AZD5153 [80] in DLBCL and BL models, have been frequently reported to have substantial in vitro and/or in vivo antitumor activities. Furthermore, ABBV-075 [75], INCB057643 [82], PLX51107, PLX2853 [83], and YLT-LL-11 [84] in DLBCL models, PFI-1 in FL models [78], and BAY1238097 in DLBCL, MCL models [85] have also shown clear anticancer activities. Overall, these studies indicated that the antitumor activities of different BETi against B-NHL were similar, mainly through inducing cell cycle arrest and apoptosis. Moreover, the gene expression signatures acquired after treatment with different BETi also highly overlapped in different lymphoma models, for instance, downregulation of E2F target, MYC target, and inhibition of the NF-κB, JAK/STAT, PI3K/AKT, MAPK signaling pathways have been most frequently reported [21, 72, 74, 75, 79, 80, 82–85].

### Preclinical studies on CREBBP/EP300 inhibitors monotherapy

Apart from the potential BETi observed in B-NHL preclinical models, non-BET BRD protein inhibitors have also been designed and developed. For instance, A-485, a CREBBP/EP300 inhibitor, has been reported to inhibit cell growth and tumor progression as well as alleviate idelalisib resistance in MCL, potentially by blocking the PI3K/AKT/mTOR and MAPK signaling pathways [87]. Another compound L002 (NSC764414), when interacted with the p300 catalytic domain’s active site, inhibited the acetylation of histones as well as suppressed STAT3 activation in lymphoma cell lines [88].

## Assessment of BRD protein inhibitors as part of combination therapies in B-NHL preclinical settings

The next section reviews the co-treatment for B-NHL using BRD protein inhibitors and other anticancer agents. The groups contain BET or CBP/p300 inhibitors combined with pathway inhibitors, epigenetic drugs, B-cell lymphoma-2 (BCL2) inhibitors, immunotherapy, chemotherapy, and other drugs (Table 3).

**Table 3:** Preclinical studies on the combinations of BRD protein inhibitors and other drugs in B-NHL

Compound	Type of agents	Combination agents	Diseases	In vitro/In vivo	Publication year	References
JQ1	BETi	EZH2 inhibitor (DZNep)	DLBCL, BL, MCL	In vitro	2013	[65]
		BTKi (ibrutinib), IKKβ inhibitor (SPC-839)	ABC-DLBCL	In vitro	2014	[21]
		BTKi (ibrutinib), HDACi (panobinostat), CDK4/6 inhibitor (palbociclib), BCL2 inhibitor (venetoclax)	MCL	Both	2015	[66]
		IMiD (lenalidomide)	PEL	Both	2016	[90]
		Anti-PD1 antibodies, Anti-4–1-BB antibodies	Eμ-Myc Lymphoma	Both	2017	[91]
		BTKi (ibrutinib)	DLBCL	In vitro	2018	[92]
		PI3Ki (BKM120), BCL2 inhibitor (venetoclax)	DLBCL, BL	In vitro	2018	[67]
		BCL2 inhibitor (venetoclax), proteasome inhibitor (bortezomib), chemotherapy agent (doxorubicin)	DLBCL	In vitro	2018	[68]
		BCL2 inhibitor (venetoclax)	DHL/THL DLBCL	In vitro	2019	[69]
		HDACi (roidepsin)	BL	In vitro	2019	[93]
OTX015	BETi	BTKi (ibrutinib), PI3Kδ inhibitor (idelalisib), mTOR inhibitor (everolimus), HDACi (SAHA, roidepsin), anti-CD20 mAb (rituximab), hypomethylating agent (decitabine), IMiD (lenalidomide), chemotherapy agents (bendamustine, doxorubicin)	DLBCL	In vitro	2015	[72]
		BTKi (ibrutinib), BCL2 inhibitor (venetoclax), CDK4/6 inhibitor (palbociclib)	MCL	In vitro	2018	[73]
		MEKi (pimasertib), ATRi (AZD6738), CHK1 inhibitor (PF00477736), WEE1 inhibitor (AZD1775), IMiD (pomalidomide), mTOR inhibitor (everolimus), BTKi (ibrutinib)	MCL	In vitro	2018	[74]
		PI3Kδ inhibitors (idelalisib, duvelisib), AKTi (MK-2206), mTOR inhibitors (everolimus, deforolimus), BTKi (ibrutinib), CDK inhibitor SNS-032	BL	In vitro	2018	[94]
		BTKi (ibrutinib), BCL2 inhibitor (venetoclax)	RT-DLBCL	Both	2021	[75]
		PI3Kα inhibitor (CYH33)	B cell Lymphoma	Both	2022	[95]
		HDACi (panobinostat, dacinostat), mTOR inhibitor (everolimus), AKTi (MK-2206), JAK2 inhibitor (TG101209)	DLBCL	In vitro	2022	[96]
CPI-203	BETi	IMiD (lenalidomide)	Bortezomib-resistant MCL	Both	2014	[76]
		BTKi (ibrutinib)	ABC-DLBCL	Both	2014	[21]
		PI3Ki (BKM120)	DLBCL, BL	Both	2018	[67]
		BCL2 inhibitor (venetoclax)	DHL	Both	2018	[77]
		CXCR4 inhibitor (IQS-01.01RS)	DLBCL	Both	2019	[97]
CPI-0610	BETi	HDACi (SAHA)	DLBCL	In vitro	2023	[79]
INCB057643	BETi	XPO1 inhibitors (selinexor, eltanexor)	HGBCL	Both	2023	[98]
		BCL2 inhibitor (venetoclax)	DLBCL/HGBCL	In vitro	2021	[82]
AZD5153	BETi	BTKi (acalabrutinib)	ABC-DLBCL	Both	2023	[99]
		SYK inhibitor (Entospletinib)	DLBCL, BL	In vitro	2022	[80]
I-BET151	BETi	IMiD (lenalidomide)	PEL	In vitro	2016	[90]
		SYK inhibitor (Entospletinib)	DLBCL, BL	In vitro	2022	[80]
I-BET762	BETi	HDACi (SAHA), BCL2 inhibitor (venetoclax)	DHL/THL DLBCL	In vitro	2019	[69]
PLX51107, PLX2853	BETi	BCL2 inhibitor (venetoclax)	DLBCL	Both	2020	[83]
BAY1238097	BETi	EZH2 inhibitors (DZNep, GSK126), mTOR inhibitor (everolimus), BTKi (ibrutinib)	DLBCL	In vitro	2017	[85]
ABBV-075	BETi	BCL2 inhibitor (venetoclax)	DLBCL	In vitro	2017	[81]
PFI-1	BETi	IMiD (lenalidomide)	PEL	In vitro	2016	[90]
RVX2135	BETi	HDACi (SAHA)	Myc-induced Murine Lymphoma	Both	2014	[86]
A-485	CBP/EP300 inhibitor	PI3Kδ inhibitor (idelalisib)	MCL	In vitro	2022	[87]
NEO2734	BET/CBP/EP300 inhibitor	BCL2 inhibitor (venetoclax)	DLBCL	Both	2020	[89]

### Combining BRD protein and signaling pathway inhibition

BET proteins interact with various signaling pathways in B-NHL, such as the JAK/STAT, PI3K/AKT/mTOR, BCR, NF-κB, and MAPK pathways. This suggests the potential of combining pathway inhibitors with BETi.

#### Combinations with BCR inhibitors

Bruton Tyrosine Kinase (BTK) is a crucial component of BCR signaling and essentially regulates cell survival and proliferation in B-cell malignancies [100]. Preclinical studies mainly focused on the combination of 1st generation BTK inhibitor, ibrutinib, with BETi. Furthermore, different BETi, such as JQ1 [21, 92], OTX015 [72, 75], CPI-203 [21], or BAY1238097 [85], have shown synergistic effects with ibrutinib in DLBCL. Moreover, AZD5153 has also shown the synergistic effect with 2nd generation BTK inhibitor acalabrutinib in ABC DLBCL cells by interrupting PAX5 and BCR signaling [99]. In BL and MCL, OTX015 co-treatment with ibrutinib resulted in a stronger anti-proliferative effect than OTX015 mono-treatment [74, 94].

Spleen tyrosine kinase (SYK) is a BCR-associated kinase that acts as a proto-oncogene in B-cell malignancies [101]. The co-treatment with BETi (AZD5153 or I-BET151) and SYK inhibitor, entospletinib, enhanced anti-proliferative effects and induced a distinct combination-specific gene expression profile in DLBCL and BL [80].

#### Combinations with PI3K/AKT/mTOR inhibitors

The co-treatment with BETi and PI3K/AKT/mTOR pathway inhibitors is also common for B-NHL. In DLBCL, BL or MCL, OTX015 combined with inhibitors of PI3K (CYH33 [95] or idelalisib [72, 94]), AKT (MK-2206 [94, 96]), or mTOR (everolimus [72, 74, 94, 96] or deforolimus [94]) have indicated greater antitumor activities than BETi mono-treatment. Moreover, BETi (JQ1 or CPI-203) also displayed a synergistic effect with PI3K inhibitor BKM120 in DLBCL and BL [67]. In addition, CBP/p300 inhibitor (A-485) combined with PI3K inhibitor (idelalisib) also enhanced the antitumor effect in MCL [87].

#### Combinations with other pathway inhibitors

In addition, OTX015 and JAK2 inhibitor (TG101209) [96], as well as JQ1 and IKKβ inhibitor (SPC-839) [21], have also indicated the synergistic effects in DLBCL. Moreover, OTX015, when given in combination with MEK inhibitor (pimasertib), had a synergistic effect on MCL [74].

### Combining BRD protein and BCL2 inhibition

BCL2 protein primarily regulates the intrinsic apoptotic pathway, and BCL2 inhibitor (venetoclax) is widely used for treating hematological tumors [102]. Moreover, in DLBCL, co-treatment with various BETi (OTX015 [75], INCB057643 [82], JQ1 [67–69], I-BET762 [69], CPI-203 [77], ABBV-075 [81], PLX51107 or PLX2853 [83]) and venetoclax have indicated the improved antitumor effect. In MCL, co-treatment with BETi (JQ1 [66] or OTX015 [73]) and venetoclax synergistically promoted apoptosis, even in the MCL cells resistant to ibrutinib. In addition, in DLBCL, pan BET/CBP/EP300 inhibitor (NEO2734) was found to be more effective than BETi or CBP/EP300 inhibitors alone, and the addition of venetoclax alleviated the resistance to NEO2734 [89].

### Combining BETi and other epigenetic drugs

#### Co-treatment with HDAC inhibitors

HDACs act as epigenetic ‘erasers’, and their inhibitors, when combined with BETi, have been commonly studied [103]. Pan HDAC inhibitor SAHA (vorinostat) was reported to act synergistically to potentiate the anti-proliferative effects of I-BET762 in DLBCL cell lines through P21 upregulation and histone acetylation [69]. Furthermore, SAHA was observed to enhance CPI-0610-mediated apoptosis in DLBCL [79]. Moreover, it also promoted Myc-induced murine lymphoma cell death and cell cycle arrest as well as synergized with BETi RVX2135 [86]. In addition, OTX015 combined with HADC inhibitors (panobinostat, dacinostat [96], vorinostat, romidepsin [72]) revealed the synergistic effects in DLBCL. Synergistic anti-proliferative effects were also observed after co-treatment with JQ1 and romidepsin in BL cells [93], as well as JQ1 and panobinostat in MCL cells [66].

#### Co-treatment with EZH2 inhibitors

Enhancer of zeste homolog 2 (EZH2) is the catalytic subunit of polycomb repressive complex 2, which catalyzes lysine 27 trimethylation on histone H3 (H3K27me3) [104]. EZH2 has been widely studied for its essential association with the cancer pathogenesis. Co-treatment of JQ1 and EZH2 inhibitor (DZNep) cooperatively inhibited MYC activation, thereby significantly restoring miR-26a expression and synergistically suppressing lymphoma growth and clonogenicity in DLBCL, BL, and MCL cells [65]. Furthermore, co-treatment with BAY1238097 and EZH2 inhibitors (DZNep or GSK126) substantially downregulated the level of H3K27me3 in DLBCL cells [85].

#### Co-treatment with DNMT inhibitors

DNMTs are essential epigenetic ‘writers’, and their inhibitors such as hypomethylating agents, are widely used in hematological malignancies [16]. The combination of hypomethylating agents and BETi has also been studied in B-NHL preclinical models, and synergism was observed with OTX015 and decitabine in DLBCL cells [72].

### Combining BETi and immunotherapy/chemotherapy

As aforementioned, immunotherapy and chemotherapy are important treatment strategies for B-NHL [9]. Targeted inhibition of the programmed cell death 1 (PD-1)/programmed cell death ligand 1 (PD-L1) axis by combining JQ1 and an anti-PD-1 antibody or an immune-stimulating anti-4-1BB antibody in mouse models with Myc-driven lymphomas showed that treatment with anti-PD1, JQ1, or anti-4-1BB alone was partially effective; however, the co-treatment resulted in more robust and sustained outcomes [91]. Moreover, the combination of OTX015 with immunomodulatory drug (IMiD) pomalidomide was found to be beneficial in MCL cell lines [74]. Furthermore, CPI-203 and IMiD lenalidomide co-treatment downregulated IRF4 and MYC expression and efficiently activated apoptosis in bortezomib-resistant MCL cells [76]. I-BET151, JQ1, and PFI-1 acted synergistically with IMiD lenalidomide in PEL [90]. Synergistic effects were also observed with OTX015 plus anti-CD20 monoclonal antibody rituximab, IMiD lenalidomide, and chemotherapy agents bendamustine and doxorubicin in DLBCL cells [72]. Another study also indicated the synergistic responses to various co-treatments using doxorubicin, bortezomib, and JQ1 in DLBCL [68].

### Combining BETi and other drugs

C-X-C chemokine receptor 4 (CXCR4) belongs to the seven-transmembrane G-protein-coupled receptors family and is closely involved in the epithelial-mesenchymal transition, invasion, angiogenesis, and maintenance of stemness of tumor cells [105]. CPI-203 and IQS-01.01RS (CXCR4 inhibitor) co-treatment reduced tumor burden by downregulating MYC and p-AKT as well as enhancing apoptosis in DLBCL [97].

Exportin-1 (XPO1) is a primary component of the nuclear export pathway and is overexpressed in almost all cancers [106]. The combination of INCB057643 with XPO1 inhibitors (selinexor and eltanexor) increased apoptosis and decreased viability in high-grade B-cell lymphoma with MYC and BCL2 rearrangements (HGBCL-DH) with or without TP53 mutations [98].

CDKs are essential regulatory factors for cell cycle progression [107]. In BL cells, co-treatment with OTX015 and CDK2/7/9 inhibitor (SNS-032) has indicated a strong synergistic effect [94]. Co-treatment with BETi (JQ1 or OTX015) and CDK4/6 inhibitor (palbociclib) induced apoptosis in ibrutinib-resistant MCL cells [66, 73]. Furthermore, co-treatment with OTX015 and inhibitors targeting cell cycle checkpoint kinases and DNA damage response [108], such as ATR (AZD6738), CHK1 (PF00477736), or WEE1 (AZD1775), also synergistically affected MCL [74].

## BRD protein degraders in B-NHL

BRD degradation represents a novel modality for targeting BRD proteins, a strategy known as proteolysis targeting chimera (PROTAC) [109]. The most common BRD protein degraders are BET degraders (Table 4). They often comprise a specific BET bonding agent (usually OTX015 or JQ1), which binds the E3 ubiquitin ligase recognition motif, thereby triggering BET proteins ubiquitination and proteasome-mediated degradation [73].

**Table 4:** Preclinical studies on BET degraders in B-NHL

BET degrader	BRD-recruiting scaffold	E3 ubiquitin ligase recognition motif	E3 ubiquitin ligase	Type of treatment	Diseases	In vitro/In vivo	Publication year	References
ARV-825	OTX015	Thalidomide	CRBN E3 ubiquitin ligase complex	Monotherapy	BL	In vitro	2015	[111]
				Monotherapy	MCL	Both	2018	[73]
ARV-711	JQ1	VHL ligand	VHL-containing complex	Monotherapy or Combined with BTKi (ibrutinib), BCL-2 inhibitor (venetoclax), CDK4/6 inhibitor (palbociclib)	MCL	Monotherapy (Both), Combination (In vitro)	2018	[73]
				Monotherapy	ABC-DLBCL	Both	2019	[110]
				Monotherapy or Combined with BTKi (ibrutinib), BCL-2 inhibitor (venetoclax)	RT-DLBCL	Both	2021	[75]
MZ1	JQ1	VHL ligand	VHL-containing complex	Monotherapy	ABC-DLBCL	Both	2021	[112]
				Monotherapy	canine DLBCL	Both	2018	[113]

BET degraders are very active in lymphoma preclinical studies [73]. For instance, ARV-825 and ARV-771 significantly inhibited cell proliferation and promoted cell apoptosis [73, 75, 110, 111], moreover, they strongly downregulated the expression of MYC in DLBCL and BL [75, 110, 111], and the expressions of CDK4/6 and cyclin D1 in MCL [73]. Co-treatment with ARV-771 and venetoclax (BCL2 inhibitor), ibrutinib (BTK inhibitor), or palbociclib (CDK4/6 inhibitor) synergistically induced apoptosis in MCL cells [73]. In addition, combining ARV-711 with ibrutinib or venetoclax exerted synergistic lethality in RT-DLBCL cells, and mice co-treated with ARV-711 and venetoclax also displayed synergistic effects in vivo [75]. Another BET degrader, MZ1, also induced apoptosis and downregulated the expressions of MYC, MYD88, NF-κB in ABC DLBCL [112]. MZ1 was also active in other tumor models, such as the canine DLBCL model, and reduced its oncogenic target gene LIN28B [113]. Furthermore, due to the efficient degradation of BET proteins, BET degraders are often more active than BETi in B-NHL, including causing more perturbations in the mRNA and protein levels, and inducing more apoptosis [73, 75, 110–112]. Additionally, CREBBP/EP300 degraders have also been designed, but they have not been researched for B-NHL [36].

Currently, BET degraders have entered clinical trials but only for solid tumors and not for hematological malignancies [109]. However, considering their significant anticancer activities in B-NHL, we hope that they will be clinically studied in the future.

## Clinical activity of BRD protein inhibitors in B-NHL

Because of the functions of BRD proteins and the preclinical studies on BRD protein inhibitors alone or in combination with other anticancer drugs, several BRD protein inhibitors, especially BETi, have entered clinical trials for B-NHL patients (Table 5).

**Table 5:** Clinical trials of BRD protein inhibitors in B-NHL

Compound	Type of agents	Treatments	Phase	Identifier	Diseases	Number of lymphoma patients	Estimated completion date	Status	Outcomes for lymphoma patients	References
OTX015	BETi	Monotherapy	I	NCT01713582	Lymphomas, MM, acute leukemia	33	2017-01-20	Completed	2 CR, 1 PR	[114]
OTX015	BETi	Monotherapy	I	NCT02698189	DLBCL, AML	9	2021-09-09	Terminated	No data	Not reported
INCB054329	BETi	Monotherapy	I/II	NCT02431260	Solid tumors, hematological malignancies	4	2018-01-31	Terminated	No ORR	[115]
INCB057643	BETi	Monotherapy	I/II	NCT02711137	Solid tumors, hematological malignancies	20	2019-02-13	Terminated	1 CR, 2 PR	[115]
CPI-0610	BETi	Monotherapy	I	NCT01949883	R/R lymphomas	64	2018-02-07	Completed	2 CR, 2 PR	[116]
RO6870810	BETi	Monotherapy	I	NCT01987362	Solid tumors, DLBCL	19	2017-10-11	Completed	2 PR	[117]
Molibresib	BETi	Monotherapy	I/II	NCT01943851	R/R hematological malignancies	18	2020-04-30	Completed	1 CR, 2 PR	[118]
FT-1101	BETi	Monotherapy	I	NCT02543879	R/R NHL, MDS/AML	10	2019-03	Completed	1 SD	[119]
CC-90010	BETi	Monotherapy	I	NCT03220347	Advanced solid tumors, R/R DLBCL	25	2026-06-15	Active, not recruiting	2 CR, 1 PR	[120]
AZD5153	BETi	Monotherapy or Combined with olaparib	I	NCT03205176	R/R lymphoma and advanced solid tumors	1	2021-04-08	Completed	Unevaluable	[121]
RO6870810	BETi	Combined with venetoclax, rituximab	I	NCT03255096	R/R DLBCL	39	2019-07-03	Completed	8 CR, 7 PR	[122]
ZEN003694	BETi	Combined with entinostat	I/II	NCT05053971	R/R lymphoma and advanced solid tumors	Unknown	2025-07-01	Recruiting	No data	Not reported
CCS1477	CBP/p300 inhibitor	Monotherapy or Combined with pomalidomide, dexamethasone, azacitidine, venetoclax	I/II	NCT04068597	Advanced hematological malignancies	Unknown	2025-06-30	Recruiting	No data	Not reported

### Clinical studies on BETi monotherapy

Clinical data of a phase I study on OTX015 in patients with lymphomas, multiple myeloma (MM), and acute leukemia (NCT01713582) indicated that in the lymphoma cases (n = 33), three DLBCL patients achieved objective responses: two complete remission (CR) and one partial response (PR) [114]. Furthermore, a clinical study on OTX015 treatment in acute myeloid leukemia (AML) and DLBCL patients was discontinued due to its limited efficacy (NCT02698189).

Moreover, in two phase I studies evaluating INCB054329 (NCT02431260) and INCB057643 (NCT02711137), 4 lymphoma cases (2 DLBCL, 1 lymphoblastic lymphoma, 1 unknown) received INCB054329 treatment, while 20 lymphoma patients (11 DLBCL, 8 FL, and 1 splenic MZL) were treated with INCB057643. The results indicated that INCB054329 had a shorter half-life and a higher interpatient pharmacokinetic variability than INCB057643. In addition, objective responses were only observed in 3 FL patients treated with INCB057643 (one CR and two PR) but not in the INCB054329 treatment group [115].

A phase I study assessed CPI-0610 among 64 R/R lymphoma patients (NCT01949883) and indicated that 4 patients had objective responses (2 CR in DLBCL, 1 PR in DLBCL, and 1 PR in FL), while 5 patients (3 FL, 1 DLBCL, and 1 nodal MZL) had prolonged stable disease (SD) [116].

Furthermore, in the phase I study on RO6870810 monotherapy in solid tumors and DLBCL patients (NCT01987362), the objective response rate (ORR) was 10.5% (n = 2 of 19) among DLBCL patients whose efficacy can be evaluable [117].

Molibresib (GSK525762) was evaluated as a single agent for treating R/R hematological malignancies, including AML, MM, and NHL, in a phase I/II clinical trial (NCT01943851). A total of 111 patients were analyzed, of which 87 were assessed in the first part and 24 were assessed in the second part. For NHL patients, three cases achieved objective responses in the first part, including one CR and two PR [118].

A phase I clinical trial of FT-1101 monotherapy (NCT02543879) in patients with R/R myelodysplastic syndrome (MDS)/AML (n = 84) and NHL (n = 10) indicated that among the evaluable NHL patients, only 1 patient receiving FT-1101 over 180 mg achieved SD [119].

CC-90010 monotherapy (NCT03220347) was assessed in patients with R/R DLBCL (n = 25) and advanced solid tumors (n = 110) in a phase I clinical trial. For DLBCL patients, 2 CR and 1 PR were observed until the observation date [120].

A phase I study investigated AZD5153 alone or in combination with olaparib (NCT03205176) in advanced solid tumors or R/R lymphoma patients, and revealed that among 49 enrolled cases, only 1 had NHL, whose efficacy may not be evaluable [121].

### Clinical studies on co-treatments with BETi and other agents

Despite the promising preclinical results of BETi monotherapy in B-NHL, only a modest activity has been observed in clinical trials, and they are not devoid of toxicities. Thus, studies to identify the potential combinations that can increase the efficacy and decrease the toxicity are still needed. At the preclinical level, the combinations of BETi with various anticancer agents, such as signaling pathway inhibitors, BCL2 inhibitors, other epigenetic agents, immunotherapy, and chemotherapy, have indicated benefits. This section reviews some combinations that have been studied clinically.

A phase Ib clinical study on the triple combination of RO6870810, venetoclax, and rituximab (NCT03255096) showed that among 39 R/R DLBCL patients, CR, PR, and SD were observed in 8, 7, and 6 cases, respectively, and indicated that the co-treatment was more efficient than RO6870810 monotherapy [122].

In the phase I/II clinical trial, the co-treatment with ZEN003694 and entinostat (HDAC inhibitor) was assessed in advanced solid tumors and R/R lymphoma patients (NCT05053971). However, the study is still recruiting and the results are non-conclusive.

### Clinical studies on CREBBP/EP300 inhibitors therapy

Besides of BETi, non-BET BRD protein inhibitors, such as CREBBP/EP300 inhibitors, have also entered clinical trials for B-NHL patients. A phase I/IIa clinical trial of CCS1477 monotherapy or co-treatment with pomalidomide, dexamethasone, azacitidine, venetoclax (NCT04068597) in patients with advanced hematological malignancies (NHL, AML, MM) is still underway.

## Clinical activity of BRD protein inhibitors in cancers

Apart from lymphoma, BRD protein inhibitors have also been used against various types of cancers in clinical trials in recent years. Among BRD protein inhibitors, BET inhibitors are widely researched in clinical trials, but part of them are still underway and without results, thus, this section only lists those that have been reported (Table 6).

**Table 6:** Recent clinical trials of BRD protein inhibitors in cancers

Compound	Type of agents	Treatments (number of patients)	Phase	Identifier	Diseases (number of patients)	Status	Efficacy outcomes	References
ABBV-075 (Mivebresib)	BETi	Monotherapy (103) or Combined with venetoclax (25)	I	NCT02391480	R/R AML (44) and solid tumors (84)	Completed	Monotherapy: 1 CRi (in AML); Combination: 2 CR, 2 PR (in AML)	[123, 124]
AZD5153	BETi	Monotherapy (34) or Combined with olaparib (15)	I	NCT03205176	R/R malignant solid tumors (48) and lymphomas (1)	Completed	Monotherapy: no ORR; Combination: 1 PR (in solid tumor)	[121]
BAY1238097	BETi	Monotherapy (8)	I	NCT02369029	Advanced solid tumors (8)	Terminated	Monotherapy: no ORR, 2 SD	[125]
BI 894999	BETi	Monotherapy (95)	I	NCT02516553	Advanced solid tumors (77) and DLBCL (18)	Completed	Monotherapy: 3 PR, 17 SD (in solid tumors); DLBCL: no discussion	[126]
BMS-986158	BETi	Monotherapy (83) or Combined with nivolumab (0)	I/IIa	NCT02419417	Advanced solid tumors (83)	Completed	Monotherapy: 2 PR, 24 SD	[127]
BMS-986378 (Trotabresib/CC-90010)	BETi	Monotherapy (135)	I	NCT03220347	Advanced solid tumors (110), R/R NHL (25)	Active, not recruiting	Monotherapy: 3 CR (1 in solid tumor, 2 in DLBCL), 2 PR (1 in solid tumor, 1 in DLBCL)	[120]
	BETi	Monotherapy (20)	I	NCT04047303	Progressive diffuse astrocytoma, anaplastic astrocytoma and glioblastoma (20)	Terminated	Monotherapy: no ORR, 7 SD	[128]
	BETi	Combined with temozolomide(TMZ) with or without radiotherapy (32)	Ib	NCT04324840	Newly diagnosed glioblastoma (32)	Terminated	Combination: 1 CR, 1 PR, 25 SD	[129]
CPI-0610 (pelabresib)	BETi	Monotherapy (64)	I	NCT01949883	R/R lymphomas (64)	Completed	Monotherapy: 2 CR, 2 PR, 5 SD	[116]
	BETi	Monotherapy (44) or Combined with ruxolitinib (84)	I/II	NCT02158858	Acute leukemia, MDS, MDS/MPN (44), and myelofibrosis (84)	Active, not recruiting	Monotherapy: 1 PR, 13 SD; Combination: 57 SVR35,46 TSS50	[130, 131]
FT-1101 (CC-95775)	BETi	Monotherapy (90) or Combined with azacitidine (4)	I	NCT02543879	R/R hematologic malignancies (94)	Completed	Monotherapy: 1 CRi, 20 SD	Abstract [119]
	BETi	Monotherapy (24)	Ib	NCT04089527	Advanced solid tumors (24)	Completed	Monotherapy: no ORR, 11 SD	Abstract [132]
GS-5829 (Alobresib)	BETi	Monotherapy (23) or Combined with exemestane or fulvestrant (10)	I	NCT02392611	Advanced solid tumors and lymphomas	Completed	Monotherapy: no ORR, 7 SD; Combination: no discussion	[133]
	BETi	Monotherapy (23) or Combined with enzalutamide (8)	Ib/II	NCT02607228	Metastatic castrate-resistant prostate cancer (31)	Terminated	Monotherapy: 1 PR, 10 SD; Combination: no ORR, 4 SD	[133]
GSK525762 (Molibresib/I-BET762)	BETi	Monotherapy (196)	I/II	NCT01587703	NUT carcinoma and other cancers (196)	Completed	Monotherapy: 4 PR, 67 SD	[134, 135]
	BETi	Monotherapy (111)	I/II	NCT01943851	R/R hematological malignancies (111)	Completed	Monotherapy: 6 CR, 7 PR, 15 SD	[118]
	BETi	Combined with fulvestrant (124)	I/II	NCT02964507	HR+/HER2− advanced or metastatic breast cancer (124)	Terminated	Combination: 16 PR, 44 SD	[136]
INCB054329	BETi	Monotherapy (69)	I/II	NCT02431260	Advanced malignancies (69)	Terminated	Monotherapy: no ORR, 21 SD	[115]
INCB057643	BETi	Monotherapy (121) or Combined with gemcitabine or paclitaxel or rucaparib or abiraterone + prednisone or ruxolitinib or azacitidine (13)	I/II	NCT02711137	Advanced malignancies (134)	Terminated	Monotherapy: 2 CR, 3 PR; Combination: 1 PR	[115]
*N*-methyl-2-pyrrolidone (NMP)	BETi	Monotherapy (13)	I	NCT02468687	R/R MM (13)	Completed	Monotherapy: no ORR, 9 SD	[137]
OTX015 (Birabresib/MK-8628)	BETi	Monotherapy (86)	I	NCT01713582	Hematological malignancies (86)	Completed	Monotherapy: 5 CR, 3 PR	[114, 138]
	BETi	Monotherapy (47)	Ib	NCT02259114	Selected advanced solid tumors (47)	Completed	Monotherapy: 3 PR, 25 SD	[139]
PLX2853	BETi	Monotherapy (44)	Ib	NCT03297424	Advanced solid tumors and lymphoma	Completed	Monotherapy: 1 CR, 2 PR, 14 SD	Abstract [140]
	BETi	Monotherapy (22)	Ib	NCT03787498	R/R AML or high-risk MDS (22)	Completed	Monotherapy: 1 CR, 2 PR, 12 SD	Abstract [141]
	BETi	Monotherapy (14) or Combined with carboplatin (23)	Ib/IIa	NCT04493619	Advanced gynecological malignancies, platinum-resistant epithelial ovarian cancer (37)	Terminated	Monotherapy: 1 PR, 5 SD; Combination: 1 PR, 9 SD	[142]
PLX51107	BETi	Monotherapy (36)	Ib/IIa	NCT02683395	Advanced solid tumors (36)	Terminated	Monotherapy: no ORR, 8 SD	Abstract [143]
	BETi	Combined with azacitidine (37)	I	NCT04022785	R/R AML, MDS (37)	Completed	Combination: 8 OR	[144]
RO6870810 (TEN-010)	BETi	Monotherapy (74)	I	NCT01987362	NUT carcinoma (8), other solid tumors (47), or DLBCL (19)	Completed	Monotherapy: 5 PR (3 in solid tumors, 2 in DLBCL), 34 SD (30 in solid tumors, 4 in DLBCL)	[117]
	BETi	Monotherapy (32)	I	NCT02308761	R/R AML, MDS (32)	Completed	Monotherapy: 1 CR, 13 SD	[145]
	BETi	Monotherapy (24)	Ib	NCT03068351	Advanced MM (24)	Completed	Monotherapy: 4 PR, 12 SD	Letter to the Editor [146]
	BETi	Combined with venetoclax and/or rituximab (39)	Ib	NCT03255096	R/R DLBCL and/or HGBCL with MYC and/or BCL2 and/or BCL6 gene rearrangements (39)	Completed	Combination: 8 CR, 7 PR, 6 SD	[122]
RO6870810 (TEN-010)	BETi	Combined with atezolizumab (36)	Ib	NCT03292172	Advanced ovarian cancer or triple negative breast cancer (36)	Terminated	Combination: 2 PR, 15 SD	Abstract [147]
TQB3617	BETi	Monotherapy (36)	I	NCT05110807	R/R hematologic malignancies (36)	Unknown	Monotherapy: 10 OR	Abstract [148]
ZEN003694 (ZEN3694)	BETi	Combined with enzalutamide (75)	Ib/IIa	NCT02711956	Metastatic castration-resistant prostate cancer (75)	Completed	Combination: 1 PR, 1 SD	[149]
	BETi	Monotherapy (0) or Combined with talazoparib (52)	IIb	NCT03901469	Triple-negative breast cancer (52)	Terminated	Combination: 2 CR, 9 PR, 7 SD	Abstract [150]
NEO2734 (EP31670)	BET/CBP/p300 inhibitor	Monotherapy	I	NCT05488548	Targeted advanced solid tumors	Recruiting	No data	Not reported
CCS1477	CBP/p300 inhibitor	Monotherapy or Combined with abiraterone acetate or enzalutamide or darolutamide or olaparib or atezolizumab	I/IIa	NCT03568656	Advanced solid/metastatic tumors	Recruiting	No data	Not reported
	CBP/p300 inhibitor	Monotherapy or Combined with (pomalidomide and dexamethasone) or (azacitidine and/or venetoclax)	I/IIa	NCT04068597	Advanced hematological malignancies	Recruiting	No data	Not reported
OPN-6602	CBP/p300 inhibitor	Monotherapy or Combined with dexamethasone	Ib	NCT06433947	R/R MM	Recruiting	No data	Not reported

### Clinical studies on BETi therapy in cancers

Clinical data of a phase I study (NCT02391480) on ABBV-075 in patients with R/R AML and solid tumors (e.g., uveal melanoma, colorectal cancer, breast cancer) indicated that in the AML cases (n = 44), one patient achieved CRi (CR with incomplete blood count recovery) in the monotherapy group, and 2 CR and 2 PR were observed in the combination group, but no objective response was observed in patients with solid tumors (n = 84) [123, 124].

A phase I study assessed AZD5153 among 48 solid tumor patients and 1 lymphoma patient (NCT03205176) and indicated that no objective response was identified in the monotherapy group, only 1 PR was identified in the combination group [121]. The phase I clinical trial of BAY1238097 monotherapy (NCT02369029) for advanced solid tumor patients (n = 8) indicated no objective response [125]. BI 894999 monotherapy (NCT02516553) was assessed in patients with advanced solid tumors (n = 77) and DLBCL (n = 18) in a phase I study, but the report only focused on the patients with solid tumors, and 3 PR were observed [126].

A phase I/IIa study on BMS-986158 (NCT02419417) enrolled only patients with solid tumors (e.g., ovarian cancer, small cell lung cancer, triple-negative breast cancer). All of the 83 patients were treated with BMS-986158 monotherapy and 2 patients experienced PR [127]. A phase I clinical trial on mono-treatment with BMS-986378 (NCT03220347) has not yet been completed, but part of the results have been published. Among the 110 patients with solid tumors, 1 CR and 1 PR were observed, and among the 25 cases with DLBCL, 2 CR and 1 PR were identified [120]. Another trial on BMS-986378 monotherapy (NCT04047303) in 20 patients with progressive diffuse astrocytoma, anaplastic astrocytoma or glioblastoma displayed no objective response [128]. However, a trial on BMS-986378 combined with temozolomide and/or radiotherapy (NCT04324840) in 32 newly diagnosed glioblastoma cases reported 1 CR and 1 PR [129].

A phase I study assessing CPI-0610 monotherapy among 64 R/R lymphoma patients (NCT01949883) indicated 2 CR and 2 PR [116]. In another phase I/II trial (NCT02158858), CPI-0610 monotherapy was assessed in patients with acute leukemia, MDS or MDS/myeloproliferative neoplasm (MPN) in the phase I study (n = 44), and CPI-0610 combined with ruxolitinib was evaluated in patients with myelofibrosis in the phase II study (n = 84). In the monotherapy group, one patient achieved PR, while in the combination group, 57 cases achieved SVR35 (spleen volume reduction of ≥ 35%), and 46 cases achieved TSS50 (total symptom score reduction of ≥ 50%) [130, 131].

GSK525762 monotherapy was assessed in 196 patients with NUT carcinoma and other cancers (NCT01587703) in a phase I/II trial, and only 4 cases experienced PR [134, 135]. GSK525762 monotherapy was also assessed in 111 patients with R/R hematological malignancies (NCT01943851) in another phase I/II trial, but 6 CR and 7 PR were observed [118]. Furthermore, a phase I/II study assessing GSK525762 combined with fulvestrant in 124 patients with HR-positive/HER2-negative advanced or metastatic breast cancer (NCT02964507) showed that 16 cases achieved PR [136].

In two phase I studies evaluating INCB054329 (NCT02431260) and INCB057643 (NCT02711137), patients with advanced malignancies were treated with INCB054329 (n = 69) or INCB057643 (n = 134). In the INCB057643 treatment group, among the 121 patients receiving INCB057643 monotherapy, 2 CR and 3 PR were observed in patients with hematological tumors, and among the 13 patients receiving co-treatments, 1 PR was observed in a patient with breast cancer receiving INCB057643 and paclitaxel. No objective response was observed in the INCB054329 treatment group [115].

A phase I trial assessing OTX015 monotherapy among 86 patients with hematological malignancies (NCT01713582) reported 5 CR and 3 PR [114, 138], while another phase I study evaluating OTX015 monotherapy in 47 patients with selected advanced solid tumors (NCT02259114) only indicated 3 PR [139].

In a phase I study evaluating RO6870810 monotherapy (NCT01987362), the objective response rates were 25% (2/8), 2% (1/47) and 11% (2/19) for patients with NUT carcinoma, other solid tumors and DLBCL, respectively [117]. A phase Ib study evaluating RO6870810 monotherapy in 32 patients with R/R AML/MDS (NCT02308761) reported 1 CR [145], and another phase Ib study assessing RO6870810 monotherapy in 24 patients with advanced MM (NCT03068351) indicated 4 PR [146]. Among 39 patients with R/R DLBCL and/or HGBCL with MYC and/or BCL2 and/or BCL6 gene rearrangements receiving co-treatment with RO6870810 and venetoclax and/or rituximab (NCT03255096), 8 CR and 7 PR were observed [122]. However, among the 36 patients with advanced ovarian cancer or triple negative breast cancer receiving co-treatment with RO6870810 and atezolizumab (NCT03292172), only 2 PR were observed [147].

### Clinical studies on CREBBP/EP300 inhibitors therapy in cancers

As reported, most current clinical advances of BETi remain in the early stages, and have shown a modest activity in hematological tumors. Besides of BETi, BET/CBP/p300 inhibitors (e.g., NEO2734) and CBP/p300 inhibitors (e.g., CCS1477, OPN-6602) have also been studied, but the trials are still recruiting and no data are available.

## Toxicity of BETi

In addition to their effectiveness, toxicity is also one of the important factors to be considered in the clinical application of BETi. Currently, clinical studies on BETi are mainly in phase I, thus, the incidence and severity of adverse events (AEs) are associated with the increase in dose and regimen [109]. A meta-analysis indicated that in BETi monotherapy, the most common and severe (grade ≥ 3) hematological AEs are anemia, thrombocytopenia and neutropenia, while the most common non-hematological AEs are nausea, decreased appetite, diarrhea, dysgeusia, fatigue, and hyperglycemia, and the most severe AE is pneumonia. However, most AEs are alleviated after drug reduction or withdrawal [151]. Furthermore, the therapeutic effects of BETi on non-neoplastic cells have also been explored, which indicated that they might also affect healthy tissues [109]. For example, JQ1 might alter the BRD4-dependent transcriptional program in cardiomyocytes, which potentially prevented heart failures associated with cardiomyocyte hypertrophy; however, it also enhanced potential cardiac AEs [152]. Therefore, it is important to monitor the functions of important organs during the treatment of BETi.

## Conclusion and perspective

In summary, BRD proteins are critical epigenetic regulatory factors and act as either tumor promoters or suppressors, thereby modulating the pathogenesis and development of B-NHL. Among the BRD proteins, the BET family is the most widely researched subfamily. BETi alone or in combination with other anticancer drugs have shown certain activities against B-NHL in preclinical and clinical studies. Furthermore, BET degraders are more active than BETi in B-NHL preclinical models. Moreover, there are relatively few clinical trials on co-treatment of B-NHL with BETi and other anticancer drugs, and BET degraders have not been assessed clinically for hematological malignancies, requiring further researches.

Additionally, researches on the mechanism of non-BET BRD proteins and the development of their related inhibitors or degraders are limited in B-NHL. Among non-BET BRD proteins, CREBBP/EP300 are the most commonly studied proteins. Currently, only CREBBP/EP300 or BET/CREBBP/EP300 inhibitors have been studied in B-NHL preclinical models, and among these inhibitors, only CCS1477 has entered a clinical trial but is still underway. In addition, CREBBP/EP300 degraders have also been designed, but they have not been studied for B-NHL. Studies on other non-BET BRD proteins are very rare and further investigation is needed. It is hoped that with the development of research, BRD proteins will become a potential therapeutic target for B-NHL.

## Data Availability

Not applicable.

## Abbreviations

ABC: Activated B-cell
AE: Adverse event
AML: Acute myeloid leukemia
ARID1A: AT-Rich Interaction Domain 1A
ASH1L: ASH1 (absent, small, or homeotic)-like
ATAD: ATPase family AAA Domain-containing protein
ATR: Ataxia telangiectasia and Rad3-related serine/threonine kinase
BAZ: Bromodomain adjacent to zinc finger domain
BCR: B-cell receptor
BCL2: B-cell lymphoma-2
BET: Bromodomain and extra-terminal domain
BETi: BET inhibitor(s)
BL: Burkitt lymphoma
B-NHL: B-cell non-Hodgkin lymphoma
BRD: Bromodomain
BRPF: Bromodomain and PHD finger-containing protein
BRWD: Bromodomain and WD repeat-containing protein
BTK: Bruton Tyrosine Kinase
CD: Chromodomain
CDK: Cyclin-dependent kinase
CDKN1A: Cyclin-dependent kinase inhibitor 1A
CECR2: Cat eye syndrome chromosome region
CHK1: Checkpoint kinase 1
CR: Complete remission
CRBN: Cereblon
CREBBP: CREB-binding protein
Cri: CR with incomplete blood count recovery
CXCR4: C-X-C chemokine receptor 4
DHL//THL: Double/triple-hit lymphoma
DLBCL: Diffuse large B-cell lymphoma
DNMT: DNA methyltransferase
EBNA1: EBV nuclear antigen 1
EBNALP: EBNA leader protein
EBV: Epstein-Barr virus
E2F: E2 factor
EP300: E1A-binding protein p300
EZH2: Enhancer of zeste homolog 2
FALZ: Fetal Alzheimer antigen
FL: Follicular lymphoma
GC: Germinal center
GCB: Germinal center B-cell
GCN5L2: General control of amino acid synthesis 5-like 2
HAT: Histone acetyltransferase
HDAC: Histone deacetylase
HDMT: Histone demethylase
HGBCL: High-grade B-cell lymphoma
HL: Hodgkin lymphoma
HMT: Histone methyltransferase
IKK: Inhibitor of kappa B kinase
IMiD: Immunomodulatory drug
IRF: Interferon regulatory factor
JAK: Janus kinase
KSHV: Kaposi's sarcoma-associated herpes virus
MAPK: Mitogen activated protein kinase
MBD: Methyl-CpG binding domain
MCL: Mantle cell lymphoma
MDS: Myelodysplastic syndrome
MLL: Myeloid/lymphoid or mixed lineage leukemia
MM: Multiple myeloma
MPN: Myeloproliferative neoplasm
mTOR: Mammalian target of rapamycin
MYC: Myelocytomatosis oncogene
MyD88: Myeloid differentiation primary response protein 88
MZL: Marginal zone lymphoma
NF-κB: Nuclear factor kappa-B
NHL: Non-Hodgkin lymphoma
ORR: Objective response rate
PAX5: Paired Box 5
PB1: Polybromo 1
PCAF: P300/CBP-associated factor
PD-1: Programmed cell death 1
PD-L1: Programmed cell death ligand 1
PEL: Pleural effusion lymphoma
PFS: Progression-free survival
PHD: Plant homeodomain
PI3K: Phosphatidylinositol-3-kinase
PKB/AKT: Protein Kinase B
PR: Partial response
PROTAC: Proteolysis targeting chimera
P-TEFb: Positive transcription elongation factor b
R/R: Relapsed/refractory
RT-DLBCL: Richter Transformation-diffuse large B-cell lymphoma
SD: Stable disease
SMARCA: SWI/SNF-related, matrix-associated, actin-dependent regulator of chromatin, subfamily A
SP: Speckled Protein
STAT: Signal transducer and activator of transcription
SVR35: Spleen volume reduction of ≥ 35%
SWI/SNF: Switch defective/sucrose non-fermentable
SYK: Spleen tyrosine kinase
TAF1: TATA box-binding protein (TBP)-associated factor 1
TET: Ten-eleven translocation
TIF1: Transcriptional intermediary factor 1
TLR: Toll-like receptor
TRIM: Tripartite motif
TSS50: Total symptom score reduction of ≥ 50%
VHL: Von-Hippel Lindau
WEE1: WEE1 G2 checkpoint kinase
XPO1: Exportin-1
ZMYND: Zinc finger, MYND domain containing proteins
